# Relationship between Awareness of Disease and Adherence to Therapeutic Regimen among Cardiac Patients

**Published:** 2015-01

**Authors:** Abbas Heydari, Elaheh Sadat Ziaee, Akram Gazrani

**Affiliations:** 1 Evidence-Based Caring Research Center, Department of Medical Surgical Nursing, School of Nursing and Midwifery, Mashhad University of Medical Sciences, Mashhad, Iran;; 2 Department of Medical Surgical Nursing, School of Nursing and Midwifery, Mashhad University of Medical Sciences, Mashhad, Iran;; 3 Department of Nursing and Midwifery, North Khorasan University of Medical Sciences, Bojnurd, Iran

**Keywords:** Awareness of the Disease, Cardiac Patients, Heart Disease, Patient Adherence

## Abstract

**Background:** Adherence to prescribed therapeutic regimen is an important element of self-care behaviors in cardiac patients. Awareness of disease may play an important role in patient adherence. Thus, this study was conducted to examine the relationship between awareness of the disease and adherence to therapeutic regimen among cardiac patients admitted to selected hospitals in Mashhad, Iran.

**Methods:** Using a descriptive correlational design, 340 patients with heart disease were selected using convenience sampling from five hospitals in Mashhad during December 2009 to May 2010. Data were collected using three questionnaires including demographic information, awareness of disease and treatment adherence (including medication, diet and physical activity). Validity and reliability of the questionnaires were examined by content validity and test-retest, respectively. Also, inter-rater reliability was examined for two data raters. Data were analyzed by SPSS software, using Spearman and Kruskal-Wallis tests.

**Results:** The results showed that only 15 percent of the study subjects had a good awareness of their disease. The majority of patients followed a good medication adherence (79 %), good diet adherence (60%), and weak physical activity adherence (61%). A significant correlation was found between knowledge and adherence to therapeutic regimen (P=0.001, r_p_=0.32).

**Conclusion:** This study showed that awareness of disease and adherence to physical activity was low in the majority of patients. It is recommended that studies should be conducted to explore effective educational programs and strategies to improve adherence to therapeutic regimen among cardiac patients.

## Introduction

Cardiovascular diseases are among the most prevalent chronic diseases and have been considered as a major cause of death worldwide.^1^^,^^2^ Also, cardiovascular diseases are the top fifth cause of disability.^3^^-^^5^ Various studies have increasingly confirmed the growth of the prevalence and incidence of cardiovascular diseases.^1^^-^^3^^,^^6^ The prevalence of this disease increases with age, from 5% at the age of 20 to 75% at the age over 75. Cardiovascular diseases are the leading cause of death in Iran as well^7^ and account for a large proportion of hospitalization.^8^

Adherence to the recommended therapeutic regimen as a self-care behavior in patients suffering from cardiac diseases is highly significant. One of the major challenges to control heart diseases is the lack of self-care behavior, such as non-adherence to medication, diet and physical activity regimens, which leads to frequent hospitalizations.^5^

It is estimated that hospitalizations can be reduced by half, if patients adhere to their therapeutic regimen.^9^ Farshidi (2004) in her study reported irregular consumption of medications as a major cause of ischemic heart disease readmission, while stress and lack of physical activity were the second cause.^10^ Malek (2004) believed that medication and diet non-adherence are the most important causes of hospitalization in patients with heart failure.^11^

Many factors cause the patients fail to adhere to their prescribed therapeutic regimen and thus aggravate their disease.^12^^-^^14^ Awareness of the disease may be a related factor for adherence to the therapeutic regimen.^15^^,^^16^ Self-management programs enable the patients to have a major role in managing their own condition, controlling their symptoms, accepting their prescribed treatment, and recognizing the time they require medical follow up.^17^

Although numerous studies have been conducted on adherence to prescribed therapeutic regimen, this issue has still remained unsolved. Furthermore, common interventions regarding this issue have not successfully increased the patients’ adherence.^18^ Therefore, this study was conducted to examine the relationship between awareness of disease and adherence to therapeutic regimen among cardiac patients admitted to hospitals affiliated to Mashhad University of Medical Sciences, Mashhad, Iran.

## Materials and Methods

This descriptive correlational study was conducted on 340 cardiac patients admitted to the cardiology department of five hospitals in Mashhad, Iran. The population of the study consisted of all patients with cardiac disease referred to the selected hospitals from December 2009 to May 2010 and met the inclusion criteria of the study. Hospitals were located in different regions of Mashhad. Various hospitals were selected because of including participants from different socioeconomic classes.

Sample size was estimated 340 participants according to a pilot study conducted on 50 patients, using the following formula:

n=[Z^ 2 ^→(1-α/2)´P(1-P)]/d^ 2 ^=[(1.96)^ 2^ (0.85) (0.15)]/0.04^ 2 ^=302(+%10)

Patients were selected using the convenience sampling with a variety of cardiac problems including heart failure, ischemic heart disease, valvular problems and cardiac arrhythmias. The inclusion criteria were hospitalization due to a type of heart disease, diagnosis of heart disease by cardiologists, Iranian nationality, living in Khorasan Razavi province and ability to answer the questions. Exclusion criteria were suffering from serious psychiatric disorders, or unwilling to respond to the questions. The ethics committee of Mashhad University of Medical Sciences approved the study (88196). 

Demographic data were collected using a researcher made questionnaire including variables such as age, gender, educational level, marital status, and medical diagnosis.

To assess the patients’ awareness of their disease, a researcher-made questionnaire consisting of eight multiple choice questions, and seven true-false questions was used. For scoring, the questions 1 to 14 were scored 1 for each correct answer and 0 for each wrong answer. For question 15 (five-choice question), choosing all the items resulted in a score of 2, choosing 2-4 items resulted in a score of 1, and 0-1 item had a score of 0. The total score was converted to a scale of 0-100, so that scores of 0-50, 50-75 and 75-100 were labeled as poor, moderate and good, respectively.

In order to evaluate adherence to therapeutic regimen, a 3-section questionnaire including diet, physical activity, and medication adherence was applied. The questionnaire was developed and scored according to Likert scale. The first section was for diet adherence and included 13 items. Some items were scored inversely. The range of total score was 0 to 46. The second section was for physical activity adherence, and included 7 items. The range of the total score was 0 to 18. The third section of the questionnaire (medication adherence) included 6 items. The range of total score was 0 to 24.

The total scores of diet, medication, and physical activity adherence were converted in a scale of 0-100. A cut off point less than 33.3 was considered as poor, 33.3-66.6 moderate and 66.6-100 as good adherence. Because the patients were unable to complete the study questionnaires, they were completed by researchers during patient’s hospitalization.

The questionnaires were developed by the authors based on previous similar studies, articles and library resources. Content validity of the questionnaires was confirmed by submitting the primary questionnaires to an expert panel. For this purpose, the questionnaires were given to 10 faculty members of Mashhad school of nursing and midwifery, who were asked to give their opinions about the questionnaires’ items. The suggestions provided by experts were reviewed by the research team and necessary corrections were made in the questionnaires.

Reliability of the questionnaires was evaluated by test-retest and inter-rater reliability methods. Test-retest reliability was calculated at 90% by administration of the questionnaire twice to a group of participants 20 days apart. Inter-rater validity was examined because the patients were unable to complete the questionnaires and the researchers were responsible for completing the patients’ questionnaires. For this, the questionnaires were filled out by 2 raters on 15 patients. The Pearson’s correlation coefficients of 0.86, 0.95 and 0.91 were obtained for diet, physical activity and medication sections of adherence questionnaire, respectively. It was 0.91 for the questionnaire of disease awareness.

Data were analyzed using SPSS software. Kruskal-Wallis and Spearman’s tests were used to examine the relationship between the study variables. 

## Results

Based on demographic data, mean of the participants’ age was 61±14.1 years; most of them (59%) were female. Summary of the patients’ demographic data is presented in Table 1.

**Table 1:** Patients’ demographic data (n=340)

**Variable**	**Number**	**Percentage**
Gender		
Female	202	59%
Male	140	41%
Age (years)		
19-30	8	2.35
31-40	19	5.58
41-50	53	15.58
51-60	79	23.23
61-70	92	27.05
71-80	78	22.35
>80	13	3.82
Education status		
Illiterate	157	45.77
Elementary	120	34.98
Junior high school	20	5.83
High school	6	1.74
Diploma	23	6.70
University degree	17	4.95
Marital status		
Single	37	10.78
Married	228	66.47
Divorced/Widow	78	22.74
Diagnosis		
Heart Failure	56	16.32
Ischemic Heart Disease	206	60.05
Valvular Heart Condition	41	11.95
Deep Vein Thrombosis	9	2.62
Cardiac Arrhythmias	31	9.03

According to the findings, only 14% of the patients were in a good level of awareness about their disease (Table 2).

**Table 2:** The patients’ awareness about their disease

**Awareness**	**Frequency (%)**
Poor	170 (50)
Moderate	122 (36)
Good	51 (14)

The majority of patients had a good level of diet adherence (60%), good medication adherence (79%), but poor physical activity adherence (61%) (Table 3).

**Table 3:** Frequency distribution of treatment adherence in patients with cardiac diseases

**Adherence**	**Diet**	**Physical Activity**	**Medication**
	**Number (%)**	**Number (%)**	**Number (%)**
Poor	12 (3)	210 (62)	31 (9)
Moderate	126 (37)	118 (34)	40 (12)
Good	205 (60)	15 (4)	272 (79)

Kruskal-Wallis test showed a significant statistical difference between the five groups of cardiac patients for awareness about the disease (P=0.018), diet adherence (P=0.046) and respecting physical activity (P=0.001). It was not significant for medication adherence (P=0.311). Results showed the maximum level of diet adherence in patients with ischemic heart disease. The maximum level of physical activity was found in patients with deep vein thrombosis and the minimum level in patients with heart failure. The maximum level of awareness about the disease was found in patients with valvular problems (Table 4).

**Table 4:** Comparison of awareness of disease and levels of adherence in different groups of cardiac patients

**Variable**	**Heart failure**	**IHD**	**Cardiac arrhythmias**	**Valvular** ** problems**	**Deep vein thrombosis**	**P value**
Awareness	3.4±7.2	3.4±8.6	4.3±8.5	3.0±8.8	2.7±10.7	0.018
Diet	6.6±31.5	6.8±32.4	7.9±29.7	8.1±29	8.0±27.7	0.046
Physical Activity	3.2±3.2	3.5±5.1	3.7±5.6	2.4±4.6	3.4±7.2	0.001
Medication	5.8±20.4	7.3±19.2	8.2±17.9	5.4±21.2	11.3±14.8	0.311

In the present study, Spearman’s correlation test showed a positive correlation between the awareness of disease and treatment adherence (P=0.001, r_p_=0.32). The study findings confirmed a statistically significant relationship between the scores of awareness of the disease and physical activity adherence in all patients with cardiac diseases (P=0.001, r_p_=0.28). There was no statistically significant relationship between the level of awareness and level of diet and medication adherence (Table 5).

**Table5:** Correlations between patients’ awareness and adherence to therapeutic regimen

**Dimension of adherence**	**r**	**P**
Medication	0.132	0.069
Physical activity adherence	0.282	0.001
Diet	0.435	0.213
Total	0.323	0.001

Level of awareness was related to the physical activity adherence in patients with ischemic heart disease (P=0.000, r_p_=0.30) as well as in patients with cardiac arrhythmias (P=0.017, r_p_=0.33). A relationship was observed between level of awareness and medical regimen adherence in patients with valvular problems (r_p_= 0.043, P=0.005). Furthermore, a significant positive relationship was found between medication and diet adherence (P=0.000, r_p_=0.29).

## Discussion

According to the study results, most of the patients had a good level of diet and medication adherence, but they were in a poor level of physical activity adherence, while half of the patients had a poor level of awareness about their disease. 

The results of the current study confirmed Khodadadi et al. (2009) and Leong et al’s (2009) findings indicating that the majority of the patients had a high level of diet adherence and poor physical activity adherence.^19^^,^^20^ The results of another study by Riegel and Carlson (2002) have also shown low physical activity adherence of patients.^21^ The patients’ view toward safety of exercise and activity limitations was a common problem reported by the patients with heart disease. It seems that lack of energy and activity intolerance could be two main reasons of patients’ non-adherence to physical activity.^21^

Lennie et al. (2008) and Van der Wal et al. (2006) in their studies demonstrated that most cardiac patients had a high level of medication adherence;^22^^,^^23^ these are consistent with the findings of present study. However, Lenhbom et al. (2009) stated in their study that non-adherence to medication regimen was common among the patients with heart disease.^24^ Albert (2008) in his study showed a poor record of medication regimens in the majority of patients.^25^ Wall (2006) demonstrated that the majority of patients had a poor level of medication and diet adherence as well as pattern of activity.^26^ On the other hand, Wall (2010) reported that the majority of patients had a high level of diet and pattern of activity adherence.^27^ Variety in patient diagnosis, symptoms and type of prescribed medications may be the cause of different results; however, our study revealed no significant relationship between the type of disease and medication adherence.

In this study, only few patients had a good level of awareness about their disease and it is consistent with the result of several studies which reported low knowledge of patients with heart disease, indicating knowledge deficit as a serious problem.^7^^,^^16^^,^^23^^,^^26^

Our study demonstrated that patients with a higher knowledge about their disease had significantly more adherence. In studies performed by Van der Wal et al. (2006), Anna Stro Mberg (2006), and Parsa-Yekta et al. (2003), similar results have been reported.^15^^,^^16^^,^^23^

Results of a qualitative study conducted by Hekmatpoo (2009) have shown insufficient patient’s training regarding diet and medicine adherence, and unhealthy lifestyle as the main obstacles in preventing readmission. All patients reported a sedentary lifestyle without exercise.^7^

In contrast with other studies, Nieuwenhuis et al. (2012) found no significant relationship between knowledge and adherence to the treatment regimen.^28^ Another study showed that only few patients who had received knowledge related to treatment regimen adherence followed their therapeutic regimen.^22^ A study by Salem et al. (2011) on 385 patients showed that 236 patients (61.3%) had moderate level of knowledge, 249 (64.7%) had poor level of adherence, and none of them had a good level of adherence.^29^

These findings are in agreement with the results of the present study indicating that the patients’ awareness has led to a change in their practice and has increased their treatment adherence. Thus, there is a need for developing methods of changing the patients’ attitude and practice in this regard. 

## Conclusion

It can be concluded that the majority of the patients had low level of awareness about their disease and physical activity adherence. It seems that patients with cardiac diseases must be recommended to do physical activity as seriously as they are advised to regularly use medication and adhere to their prescribed diet. According to the findings of this study, among three elements of adherence (medication, diet and physical activity), a significant relationship was found just between physical activity and awareness about the disease. Although awareness about the disease is a key factor for patient’s treatment adherence, non-adherence is a multidimensional problem and other related factors should be considered. Despite the current efforts on patient adherence, effective strategies to enhance treatment adherence, knowledge and perception of this group of patients should be sought. 
